# Potential trauma events and the psychological consequences for Yazidi women after ISIS captivity

**DOI:** 10.1186/s12888-020-02671-4

**Published:** 2020-05-24

**Authors:** Jan Ilhan Kizilhan, Nadine Friedl, Johanna Neumann, Leonie Traub

**Affiliations:** 1 The Institute for Transcultural Health Science, Baden-Wuerttemberg Cooperative State University, Villingen-Schwenningen, Germany; 2 Insitute for Psychotherapy and Psychotraumatology, University of Dohuk, Zakho Street 38, Dahuk, Iraq; 3 Institute for Transcultural Health Science, Baden-Wuerttemberg Cooperative State University, Schrambergerstr. 26, 78054 Villingen-Schwenningen, Germany; 4 Department of Clinical Psychology and Psychotherapy, University of Bern, Bern, Switzerland; 5 Department of Psychology, University of Vienna, Vienna, Austria

**Keywords:** PTSD, Depression, Yazidi, Prevalence, Terror

## Abstract

**Background:**

Traumatic war experiences, like the ones the Yazidi had to undergo due to the attack of the so-called Islamic State (ISIS) in August 2014, are often followed by psychological consequences such as posttraumatic stress disorder (PTSD) and depression. A more detailed analysis of such specific survivor groups is needed, to develop and implement appropriate reparation and support measures.

**Methods:**

In this study, 194 Yazidi women were examined. PTSD was assessed using the Essen Trauma Inventory (ETI) and depression using Beck’s Depression Inventory (BDI-II). The potential traumatic event (PTE) and further influential factors were compared between participants with PTSD and those with PTSD and depression, using inferential statistics.

**Results:**

Panticipants showed high rates in prevalence and comorbidity for PTSD and depression. Those diagnosed with comorbid PTSD and depression experienced a higher number of PTEs and had been captured more often and for longer compared to those with PTSD. The number of PTEs experienced was then used to predict comorbid PTSD and depression.

**Conclusion:**

Further research should consider the specific situation and the cultural expression of the Yazidi.

## Background

In August 2014, troops of the self-proclaimed *Islamic State* (ISIS) conquered areas of Northern Iraq, using extreme brutality against religious minorities such as the Yazidis. The Yazidis represent a Kurdish minority. Most of them live in Northern Iraq [1]. Their faith is monotheistic and religious traditions as well as beliefs are passed on orally. This tradition goes hand in hand with a community where people live closely together [2]. Because of their religion and their secluded settlement areas in the Sinjar Mountains, the Yazidis have been discriminated and persecuted by the neighbouring populations for centuries. For the Yazidis, the ISIS invasion was the 74th genocide in succession [1]. This perpetration is classified as a crime against humanity and as a war crime [3].

The most frequent psychological problems resulting from war events are post-traumatic stress disorders (PTSD) and depressive disorders [4, 5]. Yet, there is a lack of data with regards to the prevalence of mental health disorders among populations which find themselves in lasting and continuing displacement situations, especially in conflict-affected Middle-Eastern countries [5]. In the case of people who are forced to flee from their homeland due to natural disasters or persecution, one in ten people suffers from PTSD and one in twenty suffers from depression [6]. Rates of prevalence are estimated at even higher numbers among survivors of rape, military action, captivity, internment for ethnic or political reasons or genocide [7]. In a random sample of female survivors of the Rwandan genocide, researchers found a prevalence rate for PTSD of 58% [8] and in a random sample of women affected by sexual violence in Croatia, Bosnia and Herzegovina the prevalence rate for depression was found to be 80% [9]. In an earlier study on the Yazidis, researchers found a prevalence rate of 43% for PTSD and 40% for depression [10]. The rates of comorbidity between the two disorders are high. A systematic review showed that 44% of refugees who have resettled in a Western country and who have PTSD also suffer from depression. Moreover, 71% of refugees who fled from their homeland and who have depression also suffer from PTSD [6]. 26% of a sample of Yazidis who were displaced in Turkey, had comorbid PTSD and depression [10].

Demographic and socio-economic characteristics of displaced populations have shown to be possible moderators of mental health. Internal displacement, continuing conflict situations, and economic instability are strongly associated with poor mental health outcomes [11]. Whether people develop a mental disorder after *a potential traumatic event* (PTE) depends, among other things, on various risk factors [7] and the severity and type of the PTE(s) [12]. Depression can be seen as such a risk factor for PTSD and, vice versa, PTSD can be seen as a causal risk factor for depression. The two disorders can also be seen as independent psychological consequences linked to common risk factors and without a direct casual connection [13]. Risk factors that show a link between PTSD and depression are individual characteristics [14] like age [15], duration of education [16], having a child [17] and family status [8].

But above all, the extent [18] and the type of the traumatic event seem to play the most important role [19]. However, research findings are inconsistent [10, 20]. In the context of war-related experiences, experiencing violent attacks [21], the death of a significant person [10], torture [18], captivity [22, 23] and being a victim of a sexual attack [23] indicated a correlation with PTSD and depression. However, being in a war zone was associated with PTSD only.

Furthermore, the feeling of guilt following a traumatic event seems to be a predictor of both PTSD and depression [24]. Experiencing abuse in a violent relationship has shown to be a predictor of low self-esteem and severe symptoms of depression [25]. Quarantini and colleagues [26] mention the alleged effects that violence can have on self-confidence, self-esteem, and self-efficacy expectations. In addition to PTEs themselves, factors such as the time interval since the PTE [18] and the duration of captivity [27, 28] seem to influence the development of PTSD or depression.

In the current study, Yazidi women who were exposed to multiple PTEs have been examined with regards to the presence of PTSD, depression or comorbid PTSD and depression. The aim was to investigate differences between women with PTSD and women who have been diagnosed with PTSD and depression. The second goal of this study was to examine predictors of comorbid PTSD and depression as well as predictors of PTSD. To the best of our knowledge, this is the first study to investigate comorbid PTSD and depression and possible predictors of PTSD and depression in a sample of Yazidi women.

## Methods

### Participants

The sample consists of 190 Yazidi women from Kurdistan, Northern Iraq. They have been victims of the 2014 genocide. The average age was 32.62 years (*SD* = 10.74, range = 18–75), half of the women were younger than 30. They have been resettled in the federal state of Baden-Württemberg, Germany. This action was part of a covenant that agreed to resettle 1100 extraordinarily vulnerable Yazidi women in Germany to provide medical and psychological care. The data was collected between April and November 2015, which was before the resettlement. Contact to the women was established via the health department of the Kurdish government. Inclusion criteria were a minimum age of 18 and a diagnosis of either PTSD or comorbid PTSD and depression. At the time, all the women incorporated into the sample were living in refugee accommodation. Participants in the study reported on average 5.52 PTEs related to war (*SD* = 0.97, range = 2–7).

### Procedure

Interviews took place in Dohuk, Iraq, in a room that had been rented by the regional government. They were conducted in Kurmanji, the Yazidis’ mother tongue, and translated directly into German. All interviews were conducted by the project leader, an accredited translator for Kurdish, and psychotherapists. After the demographic data were collected, PTEs, PTSD, and depression were assessed via questionnaires.

### Measures

Demographic data such as age, duration of education, family status and motherhood as well as a brief description of PTEs were collected. The duration of captivity, whether close family members were in captivity and whether the woman had been sold on the slave market were noted, as well.

The Kurdish version of the *Essen Trauma Inventory* (ETI) [29] was used to diagnose PTSD and the PTEs in connection with war, according to the Diagnostic and Statistical Manual of Mental Disorders DSM-IV [30]. From the ETI, the following events and scenarios were selected and considered as constituting factors for a PTE: Living in a war zone (“combat mission in war or living in a war zone”), violent attack (“violent attack by a stranger, a family member or a person from the circle of friends [e.g. physically attacked, robbed, threatened with a firearm]”), the death of a person who was a close attachment figure to the individual [e.g. due to an accident, suicide, or murder], torture [e.g. sleep deprivation lasting for days, electric shocks, suffocation attempts], captivity [e.g. imprisonment due to crime, prisoner of war, hostage] and sexual attack (“as an adult, sexual attack by a stranger, a family member or person from the circle of friends [e.g. rape, attempted rape]”). The ETI also offered space for the independent detailed description of further bad events which were considered a stress factor. If a person selected *Yes, personally* or *Yes, witness* to one of the PTEs, the A1 criterion of the DSM-IV was considered as fulfilled. The tentative diagnosis of PTSD was made when the A1- and A2- criteria had been fulfilled, according to the DSM-IV and when the total score on the global scale of items for the symptoms for re-experiencing, avoidance and increased arousal reached, or exceeded, the cut-off value of 27. For the PTSD global scale, we obtained a Cronbach’s alpha of .95. The re-test reliability with 35 individuals within four weeks was .85 [29]. A reliability analysis based on this random sample showed a Cronbach’s alpha of .73 [29]. The ETI showed high cross-cultural validity.

A diagnosis of depression was made by using *Beck’s Depression Inventory*-II (BDI-II) which is a cross-culturally valid and reliable instrument for assessing depression symptoms [31]. An overall total score of 18 points or more was considered clinically relevant. Cronbach’s alpha values between .89 and .94 were obtained [31]. Because the BDI-II is allowed to be used orally with highly depressive patients and patients with reading disabilities [31], it was used during these oral interviews. In accordance with the S3 guidelines for unipolar depression, the following cut off values were defined for the BDI-II: 13–19 mild depression, 20–28 moderate depression and 29 to 63 severe depression [31]. For the present study, the cut-off value was set at 18. For this reason, only very mild forms of depression were not included. The BDI-II was translated by the interviewer.

### Data analysis

The data was processed by using *IBM SPSS Statistics 24* [32]. In the case of contradictory information or missing details about a PTE, which were discussed during the verbal anamnesis, information was taken from the case history questionnaire. The prevalence and comorbidity rates were evaluated descriptively. The *Mann-Whitney U test* [33] was used to determine whether the differences between women with PTSD and women with comorbid PTSD and depression were statistically significant for the interval-scaled variables age, duration of education, number of PTEs, time interval and duration of captivity. Differences between the category variables family status, motherhood, death of an attachment figure, torture, captivity, sexual attack, other stressful events, sale and family in captivity were checked through the use of the *chi-squared test*. The PTEs captivity, torture, and other stressful experiences did not fulfil the conditions for the chi-squared tests. For this reason, we used *Fisher’s exact test* [34]. The significance level was set according to the probability of error with α = 5%. The effect sizes for significant results in the Mann-Whitney U-test were identified through the correlation coefficients (*r)*. A value of .10 is said to be a weak effect, .10 up to .30 is a medium effect and up to .50 can be seen as a strong effect [33]. For the chi-squared tests the size of the effect was reflected through the odds ratio (*OR*) with the respective 95% confidence interval (*CI*). Likewise, the size of the effect was represented through the odds ratio (*OR*) with the respective 95% CI.

## Results

### Prevalence and comorbidity of PTSD and depression

In this study, the prevalence rate for PTSD was amounted to 97.9% and for depression to 88.1%. In 86.6% of the women, both disorders were diagnosed. 0.5% had neither disorder. 88.4% of the women with PTSD also had a diagnosis of depression, and of the ones with depression 88.0% had been diagnosed with PTSD.

### Differences between women with PTSD and women with PTSD and depression

Of the women with at least one diagnosis (*N* = 190), 22 women (11.6%) were assigned to the PTSD group and 168 women (88.4%) to the comorbid group. As can be seen in Table 1, there were no significant differences in age, length of education and the time that has passed since the worst event between women with PTSD and women with comorbid PTSD and depression. Overall, the number of years in education can be described as rather short. More than one-third of the women had no education. Neither the relationship status nor the amount of children differed significantly between these groups. Details can be taken from Table 2.

**Table 1:** Non-significant differences between age, years of education and the time interval since the worst event

	Group				
	1^a^*(M)*	2^b^*(M)*	*U*	*p*	*z*
Age	32.5	31.0	1512	.166	−1.39
Years of education^c^	3.5	5.0	1487	.122	−1.55
Time interval from the worst event^d^	210	180	1485	*.105*	*−1.62*

*Note.* Mann-Whitney U test [33]

^a^women with post-traumatic stress disorder (PTSD) according to the Essen trauma inventory

^b^women with PTSD and depression (as per Beck’s depression inventory)

^c^*N* = 190, M = 3.69, SD = 3.28, range = 0–12

^d^ Time interval from the worst event ranged from 2 to 330 days, on average the interval between the worst event and the interview was 168.20 days (*SD* = 87.12) ago

**Table 2:** Non-significant differences in relationship status and amount of children

	Total (*N* = 190)	%	χ^2^	*p*	*OR*	*95% CI*
Relationship status						
married	117	61.6	1.31^a^	.253	1.79	[0.66; 4.75]
single	60	31.6				
widowed	11	5.7				
Children^a^						
none	60	31.6	2.07^a^	.151	2.25	[0.73; 6.96]
at least one	130	68.4				

*Note.* χ^2^ = chi-squared (number of degrees of freedom, *df* = 1), calculation as per Fisher’s exact test, *OR* odds ratio

^a^Amount of children: *M* = 3, *SD* = 3.12, range = 0–13

Women with comorbid PTSD and depression (*M* = 6.00) had experienced significantly more PTEs related to war than women with PTSD (*M* = 5.00; *U* = 1023.00, *z* = − 2.79, *p* = .005, *r* = .196). The people were in captivity for an average of 192.59 days (*SD* = 107.97, range = 0–390). Around half of the women examined (52.8%) had been held captive for more than seven months. The duration of captivity revealed a significant difference between women with PTSD (*M* = 135.00) and women with PTSD and depression (*M* = 240.00). Women in the comorbid group had been in captivity for a longer time (*U* = 1313.50, *z* = − 2.22, *p* = .026, *r* = .160). 104 women (55.0%) stated that they had been sold. With regards to them, the probability of suffering from both disorders was twice as high (χ^2^ (1) = 3.15, *p* = .076, *OR =* 2.39, 95% CI [0.89, 6.42]). No difference was found between those who reported that a close family member was still in captivity (*n* = 152, 80.0%) and the others (*p* = .158, *OR* = 2.06, 95% CI [0.78, 5.49]).

Table 3 illustrates the frequency of war-related PTEs and the differences between both groups. With regards to women who had been held captive, the probability of suffering from both disorders was eight times higher. With regards to women who had suffered the death of a significant person or had experienced sexual violence the probability was twice as high as the ones who had not suffered that particular PTE. The worst events cited were captivity (62.6%), sexual attack (41.9%) and the death of a significant other (27.0%). Further PTEs that were mentioned can be taken from Table 5 in the Appendix.

**Table 3:** Comparisons of the frequency of Potential Traumatic Events (PTE) between groups with various disorders with significance evaluation and a measure of the effect size

	*n*					
	Group					
PTE	1^a^	2^b^	Total	χ^2^	*p*	*OR*
War zone						
Yes	22	168	190	–	–	–
No	–	–				
Violent Attack						
Yes	22	168	190	–	–	–
No	–	–	–			
Death of an attachment figure						
Yes	10	109	119	3.42	.065^+^	2.29
No	12	57	69			
Torture						
Yes	20	163	183	–	.145	4.08
No	2	4	6			
Captivity						
Yes	19	165	184	–	.022*	8.68
No	3	3	6			
Sexual Attack						
Yes	11	116	127	3.18	.074^+^	2.23
No	11	52	63			
Other event						
Yes	3	42	45	–	.415	1.98
No	17	120	137			

*Note.* PTE = potential traumatic event, χ^2^ = chi-squared (number of degrees of freedom, *df* = 1), calculation as per Fisher’s exact test, *OR* = odds ratio

^a^women with post-traumatic stress disorder (PTSD) according to the Essen trauma inventory

^b^women with PTSD and depression (as per Beck’s depression inventory). *p ≤ .05, ^+^*p* ≤ .10

### Predictors for PTSD and depression

The results of the binary logistic regression can be taken from Table 4. Only the number of PTEs contributed significantly to the presence of PTSD and depression (*p* = .11, *OR* = 1.85, 95% CI [088, 6.56]. For each PTE that has been experienced, the risk of suffering from depression in addition to PTSD increased to almost double. Other factors did not reach the level of significance.

**Table 4:** Binary-logistic regression with predictors for PTSD and depression, significance evaluation and measure of the effect size

	*B*	*SE*	*p*	*OR*
Constants	−1.37	1.47	.926	0.87
Number of PTEs	0.61	0.24	.011^*^	1.85

*Note. R*^*2*^ *= .07 (Cox & Snell), R*^*2*^ *= .13* (Nagelkerke)*,* Model significance value p = .002*

*N = 179,* logistic regression (LR) with predictors age, duration of education, family status, motherhood, number of PTEs, time interval, duration of captivity, sale, family in captivity, *PTSD* posttraumatic stress disorder, according to the Essen trauma inventory, depression as per Beck’s depression inventory. *p ≤ .05

## Discussion

The objectives of the present study were to examine the relations between experienced potential traumatic events (PTE) and further factors with PTSD or depression and comorbid PTSD among Yazidi women, after the ISIS attacks in 2014. In the present study, the numbers for prevalence and comorbidity were in the very high range. Women with comorbid PTSD and depression had been held captive more frequently and for a longer time and had experienced a higher number of PTEs than women with PTSD. The number of PTEs predicted the double diagnosis of PTSD and depression. The prevalence rates of 97.9% for PTSD and 88.1% for depression are higher than the numbers reported in the relevant literature [8–10]. In a previous study on the Yazidis who fled, 42.9% of the sample were reported to have been diagnosed with PTSD and 39.5% were reported to have depression [10]. The reason for the differences could be that the participants in the study of Tekin and colleagues [10] had not been held captive for more than three months. Furthermore, this study only examines women who have a higher risk of PTSD [7] and depression.

In the present study, both the frequency and the duration of captivity showed a correlation in the existence of both disorders. In a random sample of women from Croatia and Bosnia and Herzegovina who had been subjected to sexual violence and some of whom had been held captive, 14.0% had PTSD and 76.0% depression [9]. One explanation for the higher prevalence of the double diagnosis PTSD and depression among the Yazidis could be the influence of the collective, historical traumatic events entrenched in their society. Compared to individuals who have had the same experiences but did not suffer a historical trauma, the descendants of individuals who have been subjected to collective traumata show more PTSD symptoms and a generally higher risk of PTSD [35]. They identify emotionally with the traumatic events of their ancestors and report symptoms of depression such as sadness, shame, anger, shyness in taking any action, stress and low self-confidence [36]. For example, the significance which is attributed to the traumatic events plays an important role [7]. In the case of the Yazidis, identity means living together in a community with traditions and passing on their religion orally. At present, this is not possible and this represents a lasting change to their society. They do not know if it will be possible to return to their area in the near future and whether the perpetrators will be legally held accountable [1].

The number of PTEs experienced proved to be a predictor for the combined presences of PTSD and depressions. Individuals with both disorders had experienced significantly more PTEs than individuals with only PTSD. The results of the present study are consistent with results obtained from various countries and cultures [8, 18].

Individuals with both disorders had been held captive more frequently than individuals with PTSD. In a study which examined victims of kidnapping in Italy, dejectedness and hopelessness were recorded as psychological reactions to captivity [37]. This could explain the link to depression. Rather than viewing captivity per se as a PTE, the individual PTEs experienced in captivity or the accumulation of the PTEs experienced in captivity seem to have an influence. In the current study, the number of PTEs is a predictor of the comorbid presence of PTSD and depression. For this reason, the difference between both disorder groups with regards to captivity could also be the result of having experienced many PTEs in captivity. Another indication for this is that individuals with comorbid PTSD and depression in this study had spent a longer period in captivity that individuals with only PTSD. This explanation is also given in a study on those affected by sexual exploitation [27]. The authors of that study also point out that a longer period of captivity can go hand in hand with severe alienation, humiliation, hopelessness, and loss of control and this, in turn, could explain the link to depression. However, with regards to the length of captivity, in many studies there was no link between the length of captivity and depression [9, 23, 37].

Individuals with both disorders had experienced the death of a significant other, a sexual attack, or were sold more frequently. With regards to the death of an attachment figure [10] and sexual attack [27], this is consistent with previous studies. The experience of being sold was examined in this study for the first time and, therefore, presents the starting point for further research.

### Limitations

There are several limitations in the present study. First, the sample consists of a very specific group of women only without randomization. For this reason, the results cannot be generalized to other populations like other minority groups or even Yazidi women living under different circumstances. Moreover, the sample in this study showed a high level of homogeneity. Consequently, the PTEs related to living in a war zone and violent attack could not be compared, as they were experienced by all the women participating. Because of the unequal distribution of the subsamples women with PTSD (*n* = 22) and women with comorbid PTSD and depression (*n* = 168) there were, in parts, only very few participants for each set of conditions.

The interviews took place in a war zone and at the time when the information was collected, the participants were living in refugee accommodations. Some of the participants were questioned immediately after release from captivity. These circumstances might been seen as another limitation and could explain parts of the missing data. Moreover, even though all interviews were conducted by a psychotherapist with experience in working with traumatized individuals and with knowledge of the Yazidi culture, misunderstandings or mistranslations could have occurred when filling in the questionnaires. Some of the symptoms could have been unknown or unfamiliar to the Yazidi women and other symptoms relating to suffering may have been neglected.

### Future studies

Lastly, two aspects were especially noticeable with regards to the evaluation of the ETI. First, the participants had difficulties choosing “the worst event”. Apart from a few exceptions, more than one event was named as the most traumatic event. Secondly, the list of PTEs given was not sufficient to cover the things experienced by the women involved in the study.

The clinical pictures of PTSD and depression can differ significantly between cultures with regards to avoidance behaviour, deafness, nightmares and somatic symptoms [7]. To obtain a more exact picture of the specific cultural expressions of the psychological suffering of the Yazidis, a follow-up study with a more sophisticated analytical approach that allows causal conclusions about universal and culture-specific contributions should be designed and implemented. Particularly with regards to traditional societies, it seems reasonable to include a society-internal view of the expression of suffering to obtain a holistic picture of the psychological consequences of traumatic events [38]. With regards to the Yazidis, further research should specify the events in captivity. In that case, it could be ascertained whether there is a link between captivity and symptoms of depression due to individual events or due to the number of events.

## Conclusions

In spite of some limitations, this study produced promising results and, therefore, contributes to the further understanding of the psychiatric health of people globally affected by organized violence e.g. the Yazidis. As differences in cultures and in religious minorities play a role in the effects of traumatic experiences on an individual’s mental health, it is essential to gain knowledge about distinct psychosocial vulnerabilities of such groups. It is important to allow a better understanding of the effects of the aftermath of war on psychological wellbeing. As a result, interventions that do not only target mental illness but also culturally specific peculiarities can be developed [5]. With regards to the development of new therapeutic interventions for this specific population and for other survivors of mass violence, it was important to demonstrate the high prevalence of PTSD and comorbid depression in the Yazidi population. Furthermore, knowledge about the connection of PTEs and mental health among those Yazidis who survived the crimes perpetrated by ISIS is necessary. In this way, a course of action which provides the opportunity to improve their wellbeing can be developed and implemented. Demographic analyses like this one are important to define groups that are in need of special support. With the growing amount of people who need psychosocial support, vulnerable groups have to be recognized and prioritized [39].

## Data Availability

The data and materials of study are available from the corresponding author on reasonable request.

## Appendix

**Table 5:** Frequency and percentage rate of the additional worst events named (stated several times)

Event	*n*	%
Slavery	33	17.5
Massacre in the village	21	11.1
Sale	17	9.0
Forced conversion to Islam	9	4.8
Torture	7	3.7
Maltreatment	5	2.7
Threat	2	1.1
Hiding underground	1	0.5
Capture	1	0.5
Displacement/Expulsion	1	0.5
Fleeing	1	0.5
Slave labour	1	0.5
Separation from parents	1	0.5
Imprisonment	1	0.5
ISIS-induced pregnancy	1	0.5

## Abbreviations

ETI: Essen Trauma Inventory
ISIS: So called Islamic State
PTE: Potential traumatic event
PTSD: Posttraumatic stress disorder
